# Fundamental Limits of an Irreversible Heat Engine

**DOI:** 10.3390/e26121128

**Published:** 2024-12-23

**Authors:** Rui Fu

**Affiliations:** Center for Advanced Control and Smart Operations, Nanjing University, Suzhou 215163, China; ruifumath@gmail.com

**Keywords:** stochastic thermodynamics, Wasserstein distance, maximal power, optimal control strategy

## Abstract

We investigated the optimal performance of an irreversible Stirling-like heat engine described by both overdamped and underdamped models within the framework of stochastic thermodynamics. By establishing a link between energy dissipation and Wasserstein distance, we derived the upper bound of maximal power that can be delivered over a complete engine cycle for both models. Additionally, we analytically developed an optimal control strategy to achieve this upper bound of maximal power and determined the efficiency at maximal power in the overdamped scenario.

## 1. Introduction

Classical thermodynamics [1,2,3] is devoted to dealing with heat, work, and other properties in macroscopic systems. The universal laws of thermodynamics can be well applied to the study of macroscopic heat engines, where fluctuations are negligible due to the numerous internal degrees of freedom within the system. In contrast, the importance of fluctuation [4,5,6] grows as the energy scales of engines are typically reduced. This regime can be achieved when the dimensions of the system are scaled down to micrometers. Recent advances in nano-technology have brought increasing interest in thermodynamic phenomena at the microscopic scale, where the system may be away from equilibrium. Stochastic thermodynamics [7,8,9,10,11,12,13,14,15,16,17] extends classical thermodynamics to the non-equilibrium regime by incorporating the probabilistic nature of particle motion. It is rooted in using stochastic differential equations to model the random behavior of particles, allowing for quantifying energy exchanges and work during non-equilibrium thermodynamic transitions [18,19]. This framework introduces new concepts such as fluctuation theorems and thermodynamic uncertainty relations, which quantify how thermodynamic variables fluctuate around their average values, providing a more complete and accurate description of systems wherein classical thermodynamics is no longer sufficient.

In recent decades, significant progress has been made in the study of stochastic heat engines [20,21,22,23,24,25,26,27,28], both in theory and experiments, within the framework of stochastic thermodynamics. In particular, under the assumption of a low dissipation regime, Contreras-Vergara et al. [21] investigated the efficiency at maximal power of microscopic heat engines, performing Carnot-like, Stirling-like, and Ericsson-like cycles, modeled by the overdamped Langevin equation with the quadratic potential/linear control. Additionally, Dechant et al. [29] studied the optimal driving protocol of the Stirling heat engine driven by the quadratic potential in the underdamped regime, and the corresponding concrete experimental scheme for the realization of heat engines was provided. In 2012, the first experimental realization of a microscopic Stirling heat engine, wherein a single colloidal particle was subjected to a time-dependent optical laser trap and periodically coupled to two different thermal baths, was reported [28]. In the experiment, the working gas and the piston in conventional heat engines were replaced by the particle and the trapping potential, respectively. Then, in 2024, Li et al. [20] presented the experimental realization of a nano-sized stochastic Stirling heat engine involving a levitated nanoparticle subject to a harmonic optical trap coupled to a hot/cold heat bath periodically in the underdamped regime. In the present work, we theoretically investigated the optimal performance and also optimal control design of a Stirling heat engine within the framework of stochastic thermodynamics. This study is an extension of the previous study [21], focusing on the fully nonlinear regime and removing the linear control assumption both in overdamped and underdamped dynamics.

The exposition in our manuscript proceeds as follows. In Section 2, a fundamental model for Stirling-like heat engines is introduced, and the corresponding optimal control problem is formulated within the framework of stochastic thermodynamics. Systematic procedures are then applied to analyze the maximal power output and the optimal control stategy. In Section 3, the optimal control problem for output is discussed in the underdamped scenario. Finally, concluding remarks are presented in Section 4.

## 2. Problem Formulation

We begin by describing a basic model for a thermodynamic ensemble considered in this work. The dynamics of individual particles are expressed in the following stochastic differential equations.

### 2.1. Stochastic Model

We consider a thermodynamic heat engine on the microscopic scale, in which thermal fluctuations are taken into account. The evolution of the system is described by the following overdamped Langevin equation:

(1a) $$\gamma dX_{t}=-\nabla_{x}U\left(t,X_{t}\right)dt+\sqrt{2k_{B}T\left(t\right)\gamma }dB_{t},$$

where $X_{t}$, γ, and $k_{B}$ represent the location of a particle, damping coefficient, and Boltzmann constant, respectively. U(t,x) denotes a time-dependent potential that is externally controllable. Periodic function T(t) is the temperature of the heat bath at time *t*, and ${\left\{B_{t}\right\}}_{t\geq 0}$ is a standard Brownian motion. The quantity ρ(t,x) denotes the probability density to find the system in state *x* at time *t*, and satisfies the following Fokker–Planck equation:

(1b) $$\frac{\partial \rho }{\partial t}-\frac{1}{\gamma }\nabla_{x}\cdot \left[\left(\nabla_{x}U+k_{B}T\left(t\right)\nabla_{x}\log\rho \right)\rho \right]=0.$$

According to the framework of stochastic thermodynamics [10,30], the average work, at each instant time *t*, transferred to the system at the ensemble level can be expressed as follows:

(2) $$\int_{R^{n}}\frac{\partial U(t,x)}{\partial t}\rho \left(t,x\right)dx,$$

with a positive value when energy is delivered to the system, and negative when energy is drawn out. The internal energy of the system (1a), at each *t*, is defined as

(3) $$E\left(\rho ,U\right)=\int_{R^{n}}U\left(t,x\right)\rho \left(t,x\right)dx.$$

The change in internal energy between two points in time, $t_{i}$ (initial) and $t_{f}$ (final), is given by conservation of energy, which is expressed as the sum of work delivered W into the system/working medium and heat drawn out of the heat bath Q [10], i.e.,

(4) $$\Delta E=E\left(\rho \left(t_{f},.\right),U\left(t_{f},.\right)\right)-E\left(\rho \left(t_{i},.\right),U\left(t_{i},.\right)\right)=W+Q,$$

with

(5) $$W=\int_{t_{i}}^{t_{f}}\int_{R^{n}}\frac{\partial U(t,x)}{\partial t}\rho \left(t,x\right)dxdt,$$

and

(6) $$Q=\int_{t_{i}}^{t_{f}}\int_{R^{n}}\left(\;\;-\frac{1}{\gamma }{∥\nabla_{x}U\left(t,x\right)∥}^{2}\;\;+\;\;\frac{k_{B}T}{\gamma }\Delta_{x}U\left(t,x\right)\right)\rho \left(t,x\right)dxdt.$$

It is important to point out that, while ΔE depends only on the boundary values of U(t,·) and ρ(t,·), W (and as a consequence, Q as well) depends on the path/schedule

(7) $${\left\{U\left(t,\cdot \right),\rho \left(t,\cdot \right)\right\}}_{t\in [t_{i},t_{f}]}$$

that was followed. There are two other important thermodynamic quantities involved in our analysis, namely the entropy

(8) $$S\left(\rho \right)=-k_{B}\int_{R^{n}}\rho \left(t,x\right)\log\left(\rho \left(t,x\right)\right)dx,$$

and the non-equilibrium free energy [31]. Originally, the free energy is defined for systems in thermodynamic equilibrium. When the potential U(t,x) is fixed, the state distribution approaches a Boltzmann distribution

$$\rho_{B}:=\frac{1}{Z}e^{-\frac{U}{k_{B}T}},\;\;\;\;\;\;Z\;\mathrm{is}\;a\;\mathrm{partition}\;\mathrm{function},\;$$

and the relationship between internal energy $E\left(\rho_{B}\right):=\int_{R^{n}}U\left(x\right)\rho_{B}\left(x\right)dx$, Helmholtz free energy $F:=-k_{B}T\log Z$, and entropy $S\left(\rho_{B}\right):=-k_{B}\int_{R^{n}}\rho_{B}\left(x\right)\log\rho_{B}\left(x\right)dx$ in the equilibrium steady state satisfies

$$\begin{array}{c} F=E\left(\rho_{B}\right)-TS\left(\rho_{B}\right). \end{array}$$

The relationship can be extended to non-equilibrium states according to [31], defined as

(9) $$F\left(\rho ,U,T\right)=E\left(\rho ,U\right)-TS\left(\rho \right)=k_{B}T\int_{R^{n}}\rho \log\left(\frac{\rho }{\rho_{B}}\right)dx,$$

which represents relative entropy of the ensemble state with respect to the unnormalized Boltzmann distribution $\rho_{B}:=\exp\left(-U/\left(k_{B}T\right)\right)$. The change in the free energy F quantifies the upper bound of the maximum amount of work that the engine can perform in a thermodynamic process at a constant temperature *T*. In particular, the heat absorbed by the system is entirely converted into work, and the free energy change reflects the maximum available work in a reversible process. While in irreversible processes, the work performed will always be less than this upper bound due to entropy generation and the effect of other factors.

### 2.2. Thermodynamic Transitions

We here consider two types of thermodynamic processes: isothermal and isochoric transitions.

*Isothermal transitions.* The isothermal transition describes a process during which the temperature of the heat bath is constant and an outside controllable potential steers the thermodynamic state between two marginal distributions. In detail, consider the transition of the ensemble modeled by dynamics (1) with constant temperature *T*: the potential U(t,x) steers the probability density of the particle between the marginal distributions $\rho (t_{i},.)$ and $\rho (t_{f},.)$ over a time interval $\left[t_{i},t_{f}\right]$. The rate of change of free energy can be expressed as [17,32]

$$\begin{array}{cc} \frac{dF}{dt}\left(\rho ,U\right) & =\frac{d}{dt}E\left(\rho ,U\right)-T\frac{d}{dt}S\left(\rho \right)\\ \; & =\int_{R^{n}}\frac{\partial U}{\partial t}\rho \;dx+\int_{R^{n}}\left(U+k_{B}T\log\rho \right)\frac{\partial \rho }{\partial t}dx\\ \; & =\int_{R^{n}}\frac{\partial U}{\partial t}\rho \;dx-\frac{1}{\gamma }\int_{R^{n}}{∥\nabla_{x}U+k_{B}T\nabla_{x}\log\rho ∥}^{2}\rho \;dx, \end{array}$$

with the Fokker–Planck equation (1b) being used in the second equality, leading to

(10) $$\begin{array}{c} \Delta F=\int_{t_{i}}^{t_{f}}\int_{R^{n}}\frac{\partial U}{\partial t}\rho dxdt-\frac{1}{\gamma }\int_{t_{i}}^{t_{f}}\int_{R^{n}}{∥\nabla_{x}U+k_{B}T\nabla \log\left(\rho \right)∥}^{2}\rho dxdt. \end{array}$$

On the other hand, according to the extension of the second law to isothermal processes connecting non-equilibrium states [31],

$$T\Delta S_{\mathrm{total}}=W_{\mathrm{irr}}=W-\Delta F\geq 0.$$

Thus, the total entropy production can be expressed as

$$\begin{array}{c} \Delta S_{\mathrm{total}}=\int_{0}^{t_{1}}\frac{\gamma }{T}\int {∥-\frac{1}{\gamma }\left(\nabla_{x}U+k_{B}T_{h}\nabla_{x}\log\rho \right)∥}^{2}\rho dxdt, \end{array}$$

and the term $W_{\mathrm{irr}}$ quantifies the *irreversible* loss of work during transitions and is expressed as

(11) $$W_{\mathrm{irr}}=T\Delta S_{\mathrm{total}}=\frac{1}{\gamma }\int_{t_{i}}^{t_{f}}\;\;\;\int_{R^{n}}\;\;{∥\nabla_{x}U\;+\;k_{B}T\nabla \log\left(\rho \right)∥}^{2}\rho dxdt.$$

According to the first law, we have

(12) $$W=\Delta E-T\Delta S+W_{\mathrm{irr}}.$$

and the heat absorbed from the heat bath of temperature *T* is

(13) $$Q=T\Delta S-W_{\mathrm{irr}}.$$

*Isochoric transitions.* An isochoric transition is a thermodynamic process in which the volume of the closed system stays constant. As a result, the work delivered vanishes,

(14) W=0,

and the change in the internal energy satisfies

(15) ΔE=Q.

### 2.3. Thermodynamic Cycle

In this section, we will consider a complete Stirling-like cycle in finite time, which consists of two isothermal and two isochoric transitions. A periodic operation involving this type of scheduling is pursued as a method to extract work from the thermal heat bath. The following part provides a detailed description of these four branches.

**(1) Isothermal expansion:** the system undergoes an expansion process over the time interval $\left[0,t_{1}\right)$, during which the temperature of the thermal bath is held constant $T=T_{h}$. The potential function U(t,.) is designed so that the distribution of $X_{t}$ evolves from the initial distribution ρ(0,.) to a terminal distribution $\rho (t_{1},.)$. The average work input to the system is given by the definition (5) and satisfies (12)

(16) $$\begin{array}{c} W^{\left(1\right)}=\Delta E^{\left(1\right)}-T_{h}\Delta S^{\left(1\right)}+W_{\mathrm{irr}}^{\left(1\right)} \end{array}$$

where $\Delta S^{\left(1\right)}:=S\left(\rho_{t_{1}}\right)-S\left(\rho_{t_{0}}\right)$ and the irreversible work follows from (11),

(17) $$\begin{array}{c} W_{\mathrm{irr}}^{\left(1\right)}=\frac{1}{\gamma }\int_{0}^{t_{1}}\;\;\;\int_{R^{n}}\;\;{∥\nabla_{x}U\;+\;k_{B}T_{h}\nabla \log\left(\rho \right)∥}^{2}\rho dxdt. \end{array}$$

By conservation of energy, the heat input to the system is

(18) $$Q^{\left(1\right)}:=T_{h}\Delta S^{\left(1\right)}-W_{\mathrm{irr}}^{\left(1\right)}.$$

**(2) Isochoric heat removal:** The system undergoes a change in the heat bath with temperatures from $T=T_{h}$ "hot" to $T=T_{c}$ "cold" (transforming from the hot bath to a cold bath), over the time interval $\left[t_{1},t_{1}+t_{2}\right)$. During this process, the volume of the gas remains constant. As a result, the work input to the system during this transition satisfies

(19) $$W^{\left(2\right)}=0.$$

The heat is released to the low-temperature heat bath and is quantified by the change in internal energy:

$$Q^{\left(2\right)}=\Delta E^{\left(2\right)}.$$

**(3) Isothermal compression:** the system undergoes a compression process over the time interval $\left[t_{1}+t_{2},t_{1}+t_{2}+t_{3}\right)$, during which the temperature of the thermal bath is held constant $T=T_{c}$. the probability density function evolves from $\rho_{t_{2}}$ to $\rho_{t_{3}}$. Therefore,

(20) $$\begin{array}{cc} W^{\left(3\right)} & =\Delta E^{\left(3\right)}-T_{c}\Delta S^{\left(3\right)}+W_{\mathrm{irr}}^{\left(3\right)}\\ W_{\mathrm{irr}}^{\left(3\right)} & =\frac{1}{\gamma }\int_{t_{1}+t_{2}}^{t_{1}+t_{2}+t_{3}}\;\;\;\int_{R^{n}}\;\;{∥\nabla_{x}U\;+\;k_{B}T_{c}\nabla \log\left(\rho \right)∥}^{2}\rho dxdt\\ Q^{\left(3\right)} & =T_{c}\Delta S^{\left(3\right)}-W_{\mathrm{irr}}^{\left(3\right)}. \end{array}$$

**(4) Isochoric heat addition:** The last step is an isochoric process from the heat bath with temperature $T_{c}$ back to the temperature $T_{h}$ during time interval $\left[t_{1}+t_{2}+t_{3},t_{1}+t_{2}+t_{3}+t_{4}\right)$. The distribution of the ensemble is back to the starting point ρ(0,.). As before,

(21) $$\begin{array}{cc} \; & W^{\left(4\right)}=0\\ \; & Q^{\left(4\right)}=\Delta E^{\left(4\right)}. \end{array}$$

### 2.4. Power Delivered and Efficiency

For a thermodynamic heat engine, one of the quantities of interest is the total power delivered during a complete engine cycle. According to Section 2.3, the work extracted (negative of the work input) during a cyclic process can be written as

(22) $$-W=-(W^{\left(1\right)}+W^{\left(3\right)}),$$

leading to the total power delivered satisfying

(23) $$P=\frac{-W}{t_{1}+t_{2}+t_{3}+t_{4}}=-\frac{W^{\left(1\right)}+W^{\left(3\right)}}{t_{1}+t_{2}+t_{3}+t_{4}},$$

from which we see isochoric branches contribute to the delivered power solely through their duration. Thus, the optimal choice of the time span for isochoric processes gives

(24) $$t_{2}=t_{4}=0.$$

This clarifies that the instantaneous transition between the hot and cold heat baths occurs during isochoric processes, which implies that the isochoric processes are also adiabatic. On the other hand, according to the experiment realization of a stochastic Stirling heat engine [28], assuming that irreversibility only occurs in the isothermal branches is not an invalid assumption. Therefore, maximizing the power delivered during a complete engine cycle

(25) $$P=-\frac{W_{\mathrm{irr}}^{\left(1\right)}+W_{\mathrm{irr}}^{\left(3\right)}+\left(T_{c}-T_{h}\right)\Delta S^{\left(1\right)}}{t_{1}+t_{3}}$$

leads to minimizing the dissipation during isothermal transitions. It is noted that, due to the assumption on fixed densities at the ends of the isotherms, the two irreversible works can be optimized independently (which is then performed for the first one). Specifically, considering the isothermal expansion process, the optimal problem for maximizing power is casted as the following minimizing dissipation problem:

(26) minU1γ∫0t1∫Rn∥∇xU+kBTh∇log(ρ)∥2ρdxdtsubjectto..∂ρ∂t−1γ∇x·∇xU+kBTh∇xlogρρ=0       ρ(0,.):=ρ0,ρ(t1,.):=ρt1specified,t1isfixed.

It is noted that a similar problem has been discussed in the context of thermodynamics of information [31] when $t_{1}$ tends to infinity. By introducing the new "velocity" variable $\omega :=-\frac{1}{\gamma }\left(\nabla_{x}U+k_{B}T_{h}\nabla_{x}\log\rho \right)$, the optimal problem (26) can be rewritten as (here, ω can be interpreted as a new velocity variable, while the first integral in (27) is considered as accumulated kinetic energy)

(27) $$\begin{array}{cc} \; & \min_{U}\gamma \int_{0}^{t_{1}}\int_{R^{n}}{\left||\omega |\right|}^{2}\rho dxdt\\ \; & \frac{\partial \rho }{\partial t}+\nabla .\left(\rho \omega \right)=0\;\left(\mathrm{continuity}\;\mathrm{equation}\right)\\ \; & \rho \left(0,.\right)=\rho_{0},\;\rho \left(t_{f},.\right)=\rho_{f}, \end{array}$$

which is a standard optimal mass transport problem formulated by Benamou and Brenier [33,34]. Thus, the minimal value for optimal problem (26) is

(28) $$W_{\mathrm{irr}}^{(1)*}=\frac{\gamma }{t_{1}}W_{2}^{2}\left(\rho_{0},\rho_{t_{1}}\right),$$

which relates to the Wasserstein distance between probability density functions $\rho_{0},\rho_{t_{1}}$. The corresponding optimal trajectory of the distribution, denoted by $\rho^{*}\left(t,.\right)$, is the displacement interpolation between $\rho_{0}$ and $\rho_{t_{1}}$ (In brief, displacement interpolation is a continuous deformation path constructed based on the optimal mass transport map. When considering transporting a distribution $\rho_{0}$ to a distribution $\rho_{t_{1}}$, the optimal transport map provides the optimal way to move each point. Displacement interpolation then combines the movements of these individual points to form a continuous transformation process from $\rho_{0}$ to $\rho_{t_{1}}$):

(29) $$\rho^{*}\left(t,.\right)={\left(\frac{t}{t_{1}}\nabla \phi +\left(1-\frac{t}{t_{1}}\right)Id\right)}_{\#}\rho_{0},$$

where ∇ϕ denotes the optimal transport map from $\rho_{0}$ to $\rho_{t_{1}}$ such that $\rho_{t_{1}}=\nabla \phi_{\#}\rho_{0}$ (push forward of $\rho_{0}$ through ∇ϕ) (The probability density function $\rho_{1}$ obtained by the push forward of a probability density function $\rho_{0}$ through a map *T* satisfies

$$\begin{array}{c} \rho_{1}\left(y\right)=\rho_{0}\left(T^{-1}\left(y\right)\right)\left|\det(DT^{-1}\left(y\right))\right|, \end{array}$$

where $DT^{-1}$ is the Jacobian matrix of the inverse transformation $T^{-1}$), and Id defines the identity map. Moreover, up to a constant, the optimal potential function is

(30) $$U^{*}\left(t,x\right)=-\gamma \psi \left(t,x\right)-k_{B}T_{h}\log\left(\rho \left(t,x\right)\right),$$

where ψ(t,x) is the solution to the Hamilton–Jacobi–Bellman (HJB) equation $\frac{\partial \psi }{\partial t}+\frac{1}{2}{∥\nabla \psi ∥}^{2}=0$ with initial condition $\psi \left(0,x\right)=\phi \left(x\right)-\frac{1}{2}{∥x∥}^{2}$. The solution to the HJB equation is expressed by the Hopf–Lax formula [33,34]

(31) $$\psi \left(t,x\right)=\underset{y}{\inf}\left[\frac{1}{t_{1}}\psi \left(0,y\right)+\frac{{∥x-y∥}^{2}}{2t}\right].$$

Similar results are derived for the isothermal compression process. Therefore, we clarify that the total work and power extracted during one complete engine cycle satisfy

(32) $$-W\leq \left(T_{h}-T_{c}\right)\Delta S^{\left(1\right)}-\left(\frac{\gamma }{t_{1}}+\frac{\gamma }{t_{3}}\right)W_{2}^{2}\left(\rho_{0},\rho_{t_{1}}\right),$$

and

(33) $$P\leq \frac{\left(T_{h}-T_{c}\right)\Delta S^{\left(1\right)}}{t_{1}+t_{3}}-\frac{\gamma }{t_{1}t_{3}}W_{2}^{2}\left(\rho_{0},\rho_{t_{1}}\right).$$

With the optimal potential design $U^{*}\left(t,x\right)$ (30), the corresponding thermodynamic efficiency satisfies

(34) $$\eta =\frac{-W}{Q^{\left(1\right)}}=\frac{\left(T_{h}-T_{c}\right)\Delta S^{\left(1\right)}-\left(\frac{\gamma }{t_{1}}+\frac{\gamma }{t_{3}}\right)W_{2}^{2}\left(\rho_{0},\rho_{t_{1}}\right)}{T_{h}\Delta S^{\left(1\right)}-\frac{\gamma }{t_{1}}W_{2}^{2}\left(\rho_{0},\rho_{t_{1}}\right)},$$

which is consistent with the Carnot efficiency $\eta_{C}=1-\frac{T_{c}}{T_{h}}$ when time spans $t_{1},t_{3}$ for isothermal transitions tend to infinity. Next, we explore how fast the Stirling engine should operate to achieve maximal power output, which gives

(35) $$t_{1}=t_{3}=\frac{4\gamma W_{2}^{2}\left(\rho_{0},\rho_{t_{1}}\right)}{\left(T_{h}-T_{c}\right)\Delta S^{\left(1\right)}},$$

with which the maximal power output and the efficiency at maximal power are obtained as

(36) $$P^{*}=\frac{{\left(T_{h}-T_{c}\right)}^{2}{\left(\Delta S^{\left(1\right)}\right)}^{2}}{16\gamma W_{2}^{2}\left(\rho_{0},\rho_{t_{1}}\right)}$$

and

(37) $$\eta =\frac{2(T_{h}-T_{c})}{3T_{h}+T_{c}}=\frac{\eta_{C}}{2-\eta_{C}/2}$$

respectively.

## 3. Model Generalization: Underdamped Dynamics

When inertial effects are not negligible, the following dynamics have to be taken into consideration:

(38) $$\begin{array}{cc} dX_{t} & =\nu_{t}dt\\ md\nu_{t} & =-\nabla_{x}U\left(t,X_{t}\right)dt-\gamma \nu_{t}dt+\sqrt{2k_{B}T\left(t\right)\gamma }dB_{t}. \end{array}$$

The corresponding Fokker–Planck equation is

(39) $$\begin{array}{cc} \frac{\partial \rho }{\partial t}= & -\nabla .\left\{\left[\begin{array}{c} \nu \\ -\frac{1}{m}\left(\gamma \nu +\nabla_{x}U\right) \end{array}\right]\rho \right\}+\frac{\sigma^{2}}{2m^{2}}\Delta_{\nu }\rho \\ = & -\frac{\partial \rho }{\partial x}\nu +\frac{1}{m}\left[\gamma \rho +\left(\gamma \nu +\nabla_{x}U\right)\frac{\partial \rho }{\partial \nu }\right]+\frac{\sigma^{2}}{2m^{2}}\Delta_{\nu }\rho , \end{array}$$

where ρ is the function of variables t,x,ν:ρ(t,x,ν), and $\sigma^{2}:=2k_{B}T\gamma$. The work in this scenario is defined as

(40) $$W=\int_{t_{i}}^{t_{f}}\int \int \frac{\partial U(t,x)}{\partial t}\rho \left(t,x,\nu \right)dxd\nu dt.$$

Then, by following same procedures as in overdamped dynamics, we find that the derivative of the free energy satisfies

(41) $$\frac{dF}{dt}=\int \int \frac{\partial U}{\partial t}\rho \;dxd\nu -\gamma \int \int {\left||\nu +\frac{\sigma^{2}}{2m\gamma }\nabla_{\nu }\log\rho |\right|}^{2}\rho dxd\nu ,$$

then, integrating over the time domain $\left[t_{i},t_{f}\right]$, we have

(42) $$\int_{t_{i}}^{t_{f}}dF=\int_{t_{i}}^{t_{f}}\int \int \frac{\partial U}{\partial t}\rho \;dxd\nu dt-\gamma \int_{t_{i}}^{t_{f}}\int \int {\left||\nu +\frac{\sigma^{2}}{2m\gamma }\nabla_{\nu }\log\rho |\right|}^{2}\rho dxd\nu dt,$$

and

(43) $$\Delta F=W-\gamma \int_{t_{i}}^{t_{f}}\int \int {\left||\nu +\frac{\sigma^{2}}{2m\gamma }\nabla_{\nu }\log\rho |\right|}^{2}\rho dxd\nu dt.$$

Thus, the dissipation energy is given as

(44) $$W_{\mathrm{irr}}=\gamma \int_{t_{i}}^{t_{f}}\int \int {\left||\nu +\frac{\sigma^{2}}{2m\gamma }\nabla_{\nu }\log\rho |\right|}^{2}\rho dxd\nu dt.$$

Therefore, the optimal problem to maximize power output during the time interval $\left[t_{i},t_{f}\right]$ can be written as follows:

(45) $$\begin{array}{cc} \; & \min_{U}\gamma \int_{t_{i}}^{t_{f}}\int \int {\left||\nu +\frac{\sigma^{2}}{2m\gamma }\nabla_{\nu }\log\rho |\right|}^{2}\rho \;dxd\nu dt\\ \; & \frac{\partial \rho }{\partial t}+\nabla .\left\{\left[\begin{array}{c} \nu \\ -\frac{1}{m}\left(\gamma \nu +\nabla_{x}U\right)-\frac{\sigma^{2}}{2m^{2}}\nabla_{\nu }\log\rho \end{array}\right]\rho \right\}=0\\ \; & \rho \left(t_{i},.\right)=\rho_{i},\;\rho \left(t_{f},.\right)=\rho_{f}. \end{array}$$

In the underdamped case, the internal energy $E:=\frac{1}{2}m\nu^{2}+U$. According to the $It\hat{o}$’s formula,

(46) $$\begin{array}{cc} dE & =m\nu .d\nu +\frac{1}{2}md<\nu ,\nu {>}_{t}+dU\\ \; & =\nu .\left(\;-\gamma \nu -\nabla_{x}U\right)dt+\sigma \nu dB_{t}+\frac{\sigma^{2}}{2m}dt+\frac{\partial U}{\partial t}dt+\nabla_{x}U.\nu dt\\ \; & =\left(\frac{\partial U}{\partial t}-\gamma {∥\nu ∥}^{2}+\frac{\sigma^{2}}{2m}\right)dt+\sigma \nu .dB_{t}, \end{array}$$

where $<\nu ,\nu {>}_{t}$ denotes the quadratic variation of the process $\nu_{t}$. Therefore, the internal energy at the ensemble level satisfies

(47) $$dE=\int \int \left(\frac{\partial U}{\partial t}-\gamma {∥\nu ∥}^{2}+\frac{\sigma^{2}}{2m}\right)\rho dxd\nu dt.$$

On the other hand,

(48) F(ρ)=E(ρ)−TS(ρ),

together with (41), leads to

(49) $$\begin{array}{cc} \; & \gamma \int_{t_{i}}^{t_{f}}\int \int {\left||\nu +\frac{\sigma^{2}}{2m\gamma }\nabla_{\nu }\log\rho |\right|}^{2}\rho dxd\nu dt\\ = & \int_{t_{i}}^{t_{f}}\int \int \gamma {∥\nu ∥}^{2}\rho dxd\nu dt+T\Delta S-\frac{\sigma^{2}}{2m}\left(t_{f}-t_{i}\right) \end{array}$$

and

(50) $$\begin{array}{cc} W_{\mathrm{irr}} & =\gamma \int_{t_{i}}^{t_{f}}\int \int {\left||\nu +\frac{\sigma^{2}}{2m\gamma }\nabla_{\nu }\log\rho |\right|}^{2}\rho dxd\nu dt\\ \; & =\gamma \int_{t_{i}}^{t_{f}}\int \int {∥\nu ∥}^{2}\rho dxd\nu dt+T\Delta S-\frac{\sigma^{2}}{2m}\left(t_{f}-t_{i}\right). \end{array}$$

Therefore, the optimization problem (45) can be rewritten as

(51) $$\begin{array}{cc} \; & \min_{U}\int_{t_{i}}^{t_{f}}\int \int {\left||\nu |\right|}^{2}\rho dxd\nu dt\\ \; & \frac{\partial \rho }{\partial t}+\nabla .\left\{\left[\begin{array}{c} \nu \\ -\frac{1}{m}\left(\gamma \nu +\nabla_{x}U\right)-\frac{\sigma^{2}}{2m^{2}}\nabla_{\nu }\log\rho \end{array}\right]\rho \right\}=0\\ \; & \rho \left(t_{i},.\right)=\rho_{t_{i}}\;\rho \left(t_{f},.\right)=\rho_{f}, \end{array}$$

where $\Delta S=S\left(\rho_{f}\right)-S\left(\rho_{i}\right)$. For simplicity, we introduce a new variable as $f\left(t,x,\nu \right):=-\frac{1}{m}\left(\gamma \nu +\nabla_{x}U\right)-\frac{\sigma^{2}}{2m^{2}}\nabla_{\nu }\log\rho$, with which the Fokker–Planck equation (39) can be expressed as

(52) $$\frac{\partial \rho }{\partial t}+\nabla_{x}.\left(\nu \rho \right)+\nabla_{\nu }.\left(f\rho \right)=0,$$

and the corresponding optimization problem (51) can be rewritten as the following form:

(53) $$\begin{array}{cc} \; & \min_{U}\int_{t_{i}}^{t_{f}}\int \int {\left||\nu |\right|}^{2}\rho \;dxd\nu dt\\ \; & \frac{\partial \rho }{\partial t}+\nabla_{x}.\left(\nu \rho \right)+\nabla_{\nu }.\left(f\rho \right)=0\\ \; & \rho \left(t_{i},.\right)=\rho_{t_{i}},\;\rho \left(t_{f},.\right)=\rho_{f}. \end{array}$$

However, in contrast to the overdamped case, the optimal problem cannot be treated as a standard optimal mass transport problem, as it cannot be rewritten in the following form:

$$\begin{array}{cc} \; & \min_{U}\int_{t_{i}}^{t_{f}}\int \int {\left||\nu |\right|}^{2}\rho \;dxd\nu dt\\ \; & \frac{\partial \rho }{\partial t}+\nabla_{x}.\left(\nu \rho \right)+\nabla_{\nu }.\left(\nu \rho \right)=0\\ \; & \rho \left(t_{i},.\right)=\rho_{t_{i}},\;\rho \left(t_{f},.\right)=\rho_{f}. \end{array}$$

Therefore, the direct relationship between dissipation and Wasserstein distance is no longer maintained. It is clearly seen that the optimization problem (45) is highly nonlinear and conventional optimization methods do not perform well, which has been verified even for quadratic driven potential U(t,x) in [35]. It seems to be challenging to obtain an accurate theoretical solution to (45); however, a lower bound for the dissipation can be derived as follows.

Here,

(54) $$\int_{t_{i}}^{t_{f}}{\left||\nu |\right|}^{2}\rho dxd\nu dt=\int_{t_{i}}^{t_{f}}\int \rho^{x}\left(x,t\right)\int {\left||\nu |\right|}^{2}\rho \left(\nu ,t|x\right)\;d\nu dxdt,$$

where $\rho^{x}\left(x,t\right)=\int \rho \left(t,x,\nu \right)d\nu$ denotes the marginal probability density of position *x*. On the other hand, integrating the Fokker–Planck equation (52) with respect to ν, we have

(55) $$\begin{array}{cc} \; & \frac{\partial \rho^{x}\left(x,t\right)}{\partial t}+\nabla_{x}.\left(\int \nu \rho d\nu \right)\\ = & \frac{\partial \rho^{x}\left(x,t\right)}{\partial t}+\nabla_{x}.\left(\int \nu \rho \left(\nu ,t|x\right)d\nu \rho^{x}\left(x,t\right)\right)=0, \end{array}$$

leading to

(56) $$\int_{t_{i}}^{t_{f}}\int {∥\int \nu \rho (\nu ,t|x)d\nu ∥}^{2}\rho^{x}\left(x,t\right)dxdt\geq \frac{1}{t_{f}-t_{i}}W_{2}^{2}\left(\rho_{t_{i}}^{x},\rho_{t_{f}}^{x}\right).$$

Then, by applying the Cauchy–Swartz inequality with respect to velocity ν,

(57) $$\begin{array}{cc} \; & \int_{t_{i}}^{t_{f}}\int \rho^{x}\left(x,t\right)\int {\left||\nu |\right|}^{2}\rho \left(\nu ,t|x\right)\;d\nu dxdt\\ \geq & \int_{t_{i}}^{t_{f}}\int \rho^{x}\left(x,t\right){∥\int \nu \rho (\nu ,t|x)\;d\nu ∥}^{2}dxdt\\ \geq & \frac{1}{t_{f}-t_{i}}W_{2}^{2}\left(\rho_{t_{i}}^{x},\rho_{t_{f}}^{x}\right). \end{array}$$

Here, it should be noted that the first equality in (57) holds if and only if the velocity is constant, which is generally impossible in the underdamped scenario. Therefore,

(58) $$W_{\mathrm{irr}}\geq \frac{\gamma }{t_{f}-t_{i}}W_{2}^{2}\left(\rho_{t_{i}}^{x},\rho_{t_{f}}^{x}\right)+T\Delta S-\frac{k_{B}T\gamma }{m}\left(t_{f}-t_{i}\right),$$

and no optimal control strategy exists that would make the equality hold. We conclude that the total power delivered during a complete Stirling-like cycle is upper-bounded and satisfies

(59) $$\begin{array}{cc} P & =-\frac{W_{\mathrm{irr}}^{\left(1\right)}+W_{\mathrm{irr}}^{\left(3\right)}+\left(T_{c}-T_{h}\right)\Delta S^{\left(1\right)}}{t_{1}+t_{3}}\\ \; & \leq \frac{\frac{k_{B}\gamma }{m}\left(T_{h}t_{1}+T_{c}t_{3}\right)-\gamma \left(\frac{1}{t_{1}}+\frac{1}{t_{3}}\right)W_{2}^{2}\left(\rho_{0}^{x},\rho_{t_{1}}^{x}\right)}{t_{1}+t_{3}}, \end{array}$$

where the right-hand side of the inequality contains no information about the velocity of the particle. It should be emphasized that, in contrast to the overdamped case, where an optimal control force to make the equality hold in (33) always exists, this is generally not true in underdamped dynamics. The fact that the relation between velocity and position is governed by Newton’s equations of motion limits our control over the underdamped dynamics, which has been discussed in [36,37].

## 4. Concluding Remarks

In summary, we investigated the optimal performance of a Stirling-like heat engine modeled by both stochastic overdamped and underdamped dynamics within the framework of stochastic thermodynamics. Specifically, based on a connection between the dissipation minimization problem and optimal mass transport, the upper bound of maximal power that can be delivered during a complete engine cycle is derived for both overdamped and underdamped models. Furthermore, an optimal control design to achieve the upper bound of the power is obtained in the overdamped dynamics; however, this is not generally achieved by any protocol in the underdamped scenario. It is hoped that the present work will serve to provide insights for the design of future micro-machines.

## Data Availability

No new data were created.

## Symbols

The following symbols are used in this manuscript:

$B_{t}$	Brownian motion
E	internal energy
F	free energy
$k_{B}$	Boltzmann constant
*m*	mass
*P*	power
Q	heat
S(ρ)	entropy
*t*	time
*T*	temperature
U(t,x)	potential
W	work
$W_{\mathrm{irr}}$	dissipation energy
$W_{2}\left(\cdot ,\cdot \right)$	Wasserstein metric
$X_{t}$	position of particle
γ	damping coefficient
η	efficiency
$\nu_{t}$	velocity of particle
ρ(t,x),ρ(t,x,ν)	probability density of the particle
